# Reliability of Cell-Free DNA and Targeted NGS in Predicting Chromosomal Abnormalities of Patients With Myeloid Neoplasms

**DOI:** 10.3389/fonc.2022.923809

**Published:** 2022-06-14

**Authors:** Andrew Ip, Alexandra Della Pia, Gee Youn (Geeny) Kim, Jason Lofters, James Behrmann, Dylon Patel, Simone Kats, Jeffrey Justin Estella, Ivan De Dios, Wanlong Ma, Andrew L. Pecora, Andre H. Goy, Jamie Koprivnikar, James K. McCloskey, Maher Albitar

**Affiliations:** ^1^Hackensack University Medical Center, Oncology, Hackensack, NJ, United States; ^2^John Theurer Cancer Center, Hackensack University Medical Center, Hackensack, NJ, United States; ^3^Hackensack Meridian School of Medicine, Oncology, Nutley, NJ, United States; ^4^Ernest Mario School of Pharmacy at Rutgers University, Department of Pharmacy Practice and Administration, Piscataway, NJ, United States; ^5^Englewood Health Internal Medicine Residency Program, Englewood, NJ, United States; ^6^Genomic Testing Cooperative, Hematology, Irvine, CA, United States

**Keywords:** myeloid, NGS, cytogenetics, cfDNA, CNVs, liquid biopsy, chromosomal abnormalities

## Abstract

**Introduction:**

Cytogenetic analysis is important for stratifying patients with various neoplasms. We explored the use of targeted next generation sequencing (NGS) in detecting chromosomal structural abnormalities or copy number variations (CNVs) in patients with myeloid neoplasms.

**Methods:**

Plasma cell-free DNA (cfDNA) from 2821 myeloid or lymphoid neoplasm patients were collected. cfDNA was sequenced using a 275 gene panel. CNVkit software was used for analyzing and visualizing CNVs. Cytogenetic data from corresponding bone marrow (BM) samples was available on 89 myeloid samples.

**Results:**

Of the 2821 samples, 1539 (54.5%) showed evidence of mutations consistent with the presence of neoplastic clones in circulation. Of these 1539 samples, 906 (59%) showed abnormalities associated with myeloid neoplasms and 633 (41%) with lymphoid neoplasms. Chromosomal structural abnormalities in cfDNA were detected in 146 (16%) myeloid samples and 76 (12%) lymphoid samples. Upon comparison of the myeloid samples with 89 BM patients, NGS testing was able to reliably detect chromosomal gain or loss, except for fusion abnormalities. When cytogenetic abnormalities were classified according to prognostic classes, there was a complete (100%) concordance between cfDNA NGS data and cytogenetic data.

**Conclusions:**

This data shows that liquid biopsy using targeted NGS is reliable in detecting chromosomal structural abnormalities in myeloid neoplasms. In specific circumstances, targeted NGS may be reliable and efficient to provide adequate information without the need for BM biopsy considering broad mutation profiling can be obtained through adequate sequencing within the same test. Overall, this study supports the use of liquid biopsy for early diagnosis and monitoring of patients with myeloid neoplasms.

## Introduction

Chromosomal variations play a major role in the diagnosis, prognosis, and selection of therapy in hematologic neoplasms (1, 2). Cytogenetic studies are recommended for a majority of myeloid or lymphoid neoplasms and are integrated into the clinical care of patients with these diseases (3, 4). For example, the European LeukemiaNet (ELN) recommendations for diagnosis and management of acute myeloid leukemia (AML) state conventional cytogenetic analysis remains mandatory in the evaluation of suspected AML, and these guidelines provide risk stratification recommendations based on the results of cytogenetic studies (5). However, cytogenetic analysis has considerable limitations as these studies are more costly, require fresh viable cells for culturing, need to be manually performed by an expert, and have a turnaround time of approximately two weeks for results (6). Therefore, there has been growing interest in alternative and less invasive methods to determine chromosomal variations for patients with hematologic neoplasms.

One widely accepted method as an adjunct to cytogenetic analysis is fluorescence *in situ* hybridization (FISH) studies, which comparatively have a shorter turnaround time to results of approximately 2 days, are more cost effective, can be prepared in formalin-fixed paraffin-embedded (FFPE) samples, and eliminates the need for tissue cultures (6, 7). However, FISH studies can only test for predetermined chromosomal abnormalities one at a time and are unable to identify other non-targeted chromosomal variations (6). Recent advances in high-throughput genomic technologies have allowed for broader evaluation of chromosomal abnormalities using arrays. Array technology is another alternative that offers wider genome coverage with higher resolution, but this technology has its pitfalls as it still requires a significant quantity of samples to produce accurate results (7). Another method is whole-genome sequencing (WGS), which has been proven reliable in detecting various chromosomal abnormalities including amplifications, copy number variations (CNVs), uniparental disomy, mosaicism, small indels and single nucleotide variations (SNVs) (4, 8, 9). Unfortunately, the high costs of whole-genome sequencing restrict its use in favor of targeted sequencing panels. Targeted sequencing has the advantage of being a more practical, feasible, and cost-effective approach for analyzing cell-free DNA (cfDNA) in liquid biopsies but is limited to detecting only point mutations and indels (10).

Next-generation sequencing (NGS) is a novel method that is increasingly being implemented to evaluate chromosomal structural abnormalities and has the advantage of being applied to both targeted and untargeted panels to find genome-wide DNA variations (7, 11). Recent studies have demonstrated that NGS provides a high level of resolution when searching for small numerical aberrations and loss of heterozygosity events (12). With increasing utilization of NGS for diagnostic and prognostic monitoring, there is a continued need for data to support its accuracy in detecting chromosomal abnormalities in liquid biopsies of patients with hematologic neoplasms. The clinical reliability of liquid biopsies in detecting SNVs has been established and accepted in certain solid tumors and hematologic neoplasms (13, 14). Even in myeloid neoplasms, where frequently peripheral blood cells can be used for detecting molecular abnormalities, liquid biopsy cfDNA has been shown to be as sensitive as bone marrow (BM) DNA in detecting molecular abnormalities (15–18). Noninvasive Prenatal Screening (NIPS) is currently an acceptable approach for prenatal screening to detect chromosomal structural abnormalities (3). NIPS is used mainly for detecting trisomies in specific chromosomes but is not able to identify small size chromosomal abnormalities. Thus, the clinical relevance of detecting chromosomal structural abnormalities in cancer patients has not been addressed adequately. These methods may have clinical relevance and applicability if incorporated into the routine diagnosis and management of patients with hematologic neoplasms.

Here, we aim to explore the potential of using NGS technology to evaluate chromosomal gain or loss in liquid biopsies of patients with myeloid neoplasms.

## Materials and Methods

### Patients

We obtained 2821 cfDNA peripheral blood samples from patients at the John Theurer Cancer Center and Hackensack University Medical Center between March 2020 and September 2021. Patients included in this study had either confirmed or expected lymphoid or myeloid neoplasms. Some of these samples were obtained at initial diagnosis while others were measured during follow-up and minimal residual disease (MRD) detection. The trial was conducted under the International Conference on Harmonization Good Clinical Practice guidelines and according to the Declaration of Helsinki. Institutional Review Board (IRB) approval was obtained under Hackensack Meridian Health IRB Study# Pro2020–0487. The requirement for patient informed consent (verbal or written) was waived by the IRB as this project represented a non-interventional study utilizing routinely collected data for secondary research purposes.

### Sample Collection and cfDNA Isolation

We extracted cfDNA from 2821 peripheral blood samples using the Apostle MiniMax™ High Efficiency cfDNA Isolation Kit (San Jose, CA). These peripheral blood samples were collected in EDTA anticoagulant over approximately 18 months. DNA was extracted from separated plasma within 48 hours of collection. We tested these samples for the presence of circulating tumor DNA (ctDNA).

### NGS for Mutation and Cytogenetic Analysis

The DNA was extracted and portioned into 100 ng samples to be used for sequencing. The design of the targeted NGS panel used in our study identified chromosomal structural abnormalities, which included chromosomal gain or loss but not chromosomal translocations. The library for targeted 275 gene sequencing is based on Single Primer Extension (SPE) chemistry. The DNA sequencing includes all coding exons of the 275 genes (see **Figure S1 in Supplement**). For each exon, approximately 50 intronic nucleotides were also sequenced. Genomic DNA samples underwent end repair, received A-tails and unique molecular identifiers (UMIs), and were sample indexed. Target enrichment was performed post-UMI assignment to ensure that DNA molecules containing UMIs were sufficiently enriched in the sequenced library. For enrichment, ligated DNA molecules were subjected to several cycles of targeted polymerase chain reaction (PCR) using one region-specific primer and one universal primer complementary to the adapter. A universal PCR was ultimately carried out to amplify the library and add platform-specific adapter sequences and additional sample indices. The sequencing was conducted using the Illumina NextSeq 550 or NovaSeq 6000 instruments.

### CNVKit for Copy Number Detection

The CNVkit software was implemented to evaluate CNVs in the analyzed samples (19). Notably, the software takes advantage of both on- and off-target sequencing reads, compares binned read depths in on- and off-target regions to pooled normal reference, and estimates the copy number at various resolutions. Log2 change from a pool of normal control of ±0.4 was used as an indication of chromosomal gain or loss. A log2 of -1.50 was used as the cut-off for considering homozygous loss. When a loss and a gain were noted on the same arm, the abnormality closer to the centromere was considered “proximal” and the one farther from the centromere was considered “distal.”

## Results

### Liquid Biopsy Positivity Rate for Detecting Chromosomal Structural Abnormalities

Of the tested 2821 samples, 1539 (54.5%) showed evidence of mutations consistent with the presence of neoplastic clones. Of the 1539 samples with mutations, 906 (59%) had abnormalities associated with myeloid neoplasms and 633 (41%) had abnormalities associated with lymphoid neoplasms. The median variant allele frequency (VAF) was 8.09% (range: 0.002%-99.55%). Of the 906 myeloid cases, 146 (16%) unique patient samples showed chromosomal structural abnormalities. Of the 633 lymphoid neoplastic cases, 76 (12%) unique patient samples had chromosomal abnormalities detected in cfDNA.

All cases with chromosomal structural abnormalities showed mutations in one or more genes, except for two samples that showed chromosomal structural abnormalities but no detectable mutations. One myeloid case showed a sole 5q deletion without any other mutations. This case was diagnosed as an isolated 5q deletion syndrome based on cfDNA analysis, which was confirmed by BM morphology and cytogenetic studies. The second case was a patient diagnosed with chronic lymphocytic leukemia (CLL) having shown no evidence of any mutations; however, trisomy 12 was detected on cfDNA analysis.

### Patients With Concomitant Cytogenetic Data

Peripheral blood samples from 144 patients with suspected or confirmed myeloid neoplasms were used to extract cfDNA for NGS testing. The median age was 68.5 years (range: 24-96), and 39% were female. Of those with known hematologic neoplasms, there were 22% diagnosed with AML, 34% with myelodysplastic syndrome (MDS), and 44% with myeloproliferative neoplasms (MPN) (**Table 1**). These peripheral blood samples showed mutations in genes typically seen in myeloid neoplasms including: DNMT3A (9%), FLT3 (5%), TP53 (9%), NPM1 (4%), IDH1/2 (4%), ASXL1 (4%), KRAS/NRAS (6%), U2AF1 (4%) and others.

**Table 1:** Patient characteristics.

Characteristic	Patientsn=144, n (%)
Age, years, median (range)	68.5 (24-96)
Male	88 (61)
Diagnosis Acute myeloid leukemia (AML) Myelodysplastic syndrome (MDS) Myeloproliferative neoplasms (MPN)	31 (22) 49 (34) 64 (44)

### Sensitivity of NGS in Detecting Chromosomal Structural Abnormalities

In order to evaluate the sensitivity of liquid biopsies in detecting chromosomal abnormalities, we evaluated the VAF in the cases with demonstrable chromosomal abnormalities in the cfDNA. The VAF varied between 0.002% and 99.55% depending on the type of mutation and the expected heterogeneity within the same sample.

**Figure 1:**
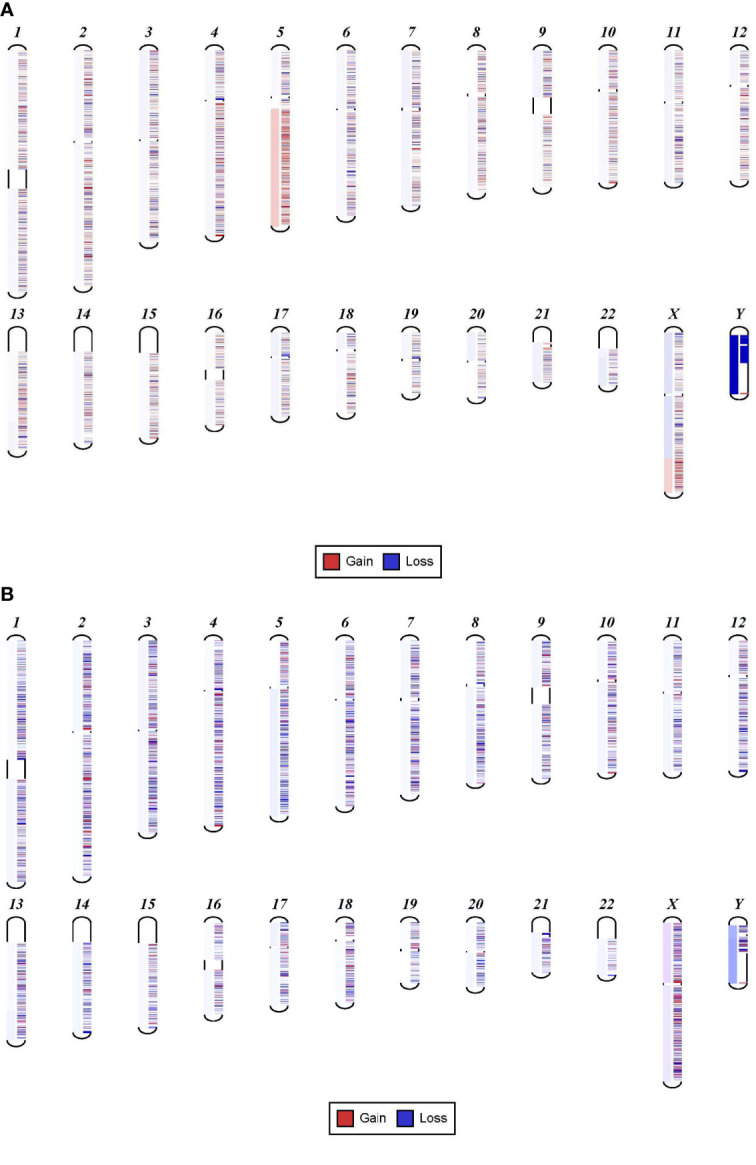
Sensitivity of NGS in detecting chromosomal gain or loss. **(A)** Shows 5q gain in a sample with FLT3 mutation detected at 13%. **(B)** Shows 5q deletion that is very faint that can be easily missed. This sample has a mutation in TP53 at 8%.

We found that samples with VAF detected at 13% or higher were associated with clear, detectable chromosomal structural abnormalities. For example, one sample shows a clear 5q gain and a mutation in FLT3 at 13% (**Figure 1A**). In contrast, a different sample with a mutation in TP53 detected at 8% shows a 5q deletion that is very faint and can be easily missed (**Figure 1B**). Levels less than 8% VAF in a sample can be considered below the level necessary for detecting specific CNVs. The exception to this lower limit is when the neoplasm is driven by the chromosomal abnormalities, such as in isolated 5q deletion syndrome and rare cases of other neoplasms or neoplasms with fusion genes.

### Correlation Between NGS Chromosomal Structural Analysis and BM Cytogenetic Studies

**Figure 2:**
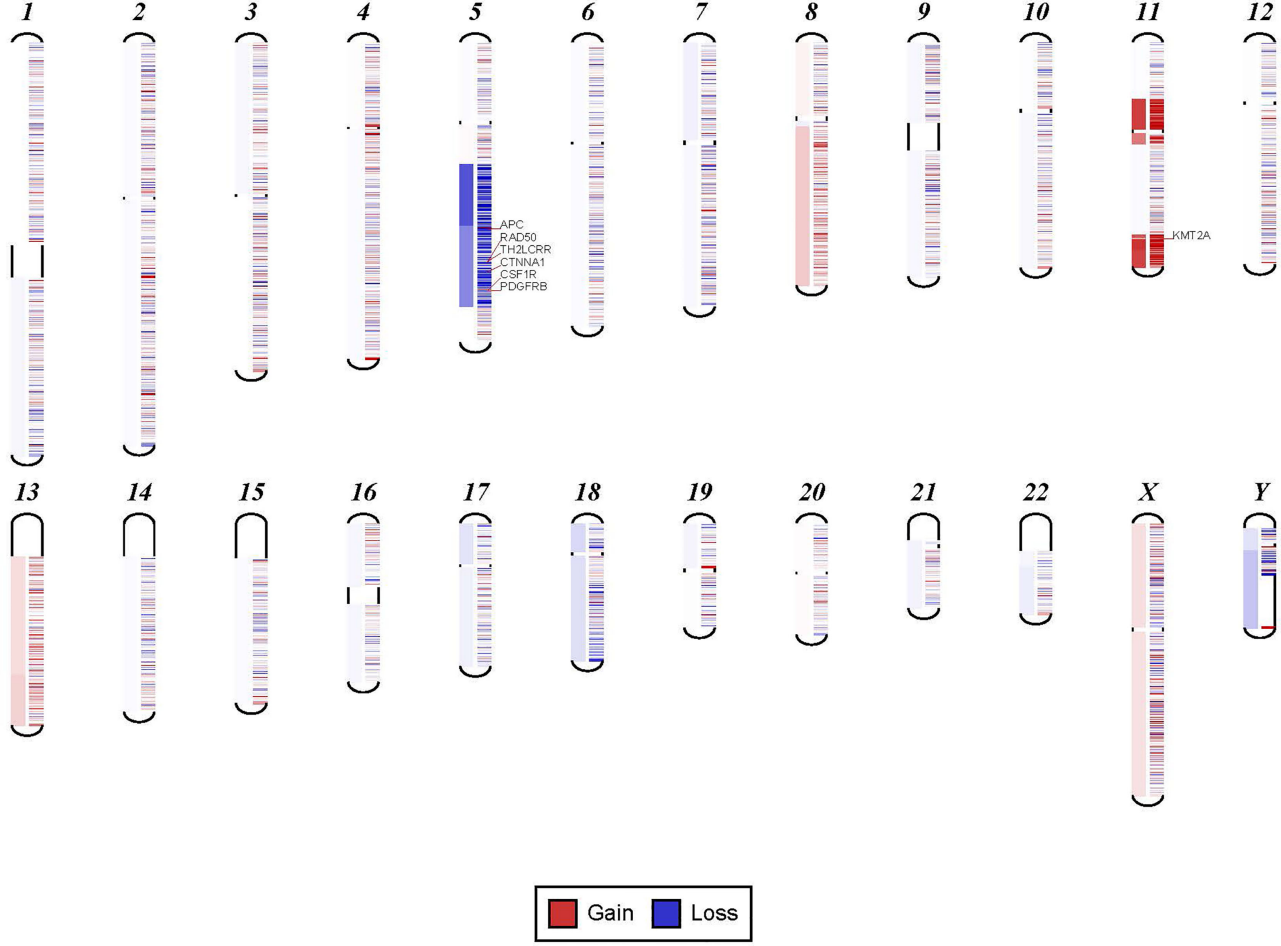
Example for discrepancy in cytogenetic description between NGS findings. The findings visualized here by NGS were described by cytogenetic analysis as “48,XY,del(5)(q13q33),+8,+mar[20].” NGS showed the 5q deletion and 8q gain and gene amplification involving chromosome 11, particularly KMT2A gene, which is most likely reported as a marker chromosome. In addition, trisomy 13 was noted by NGS that was not reported by cytogenetics.

**Figure 3:**
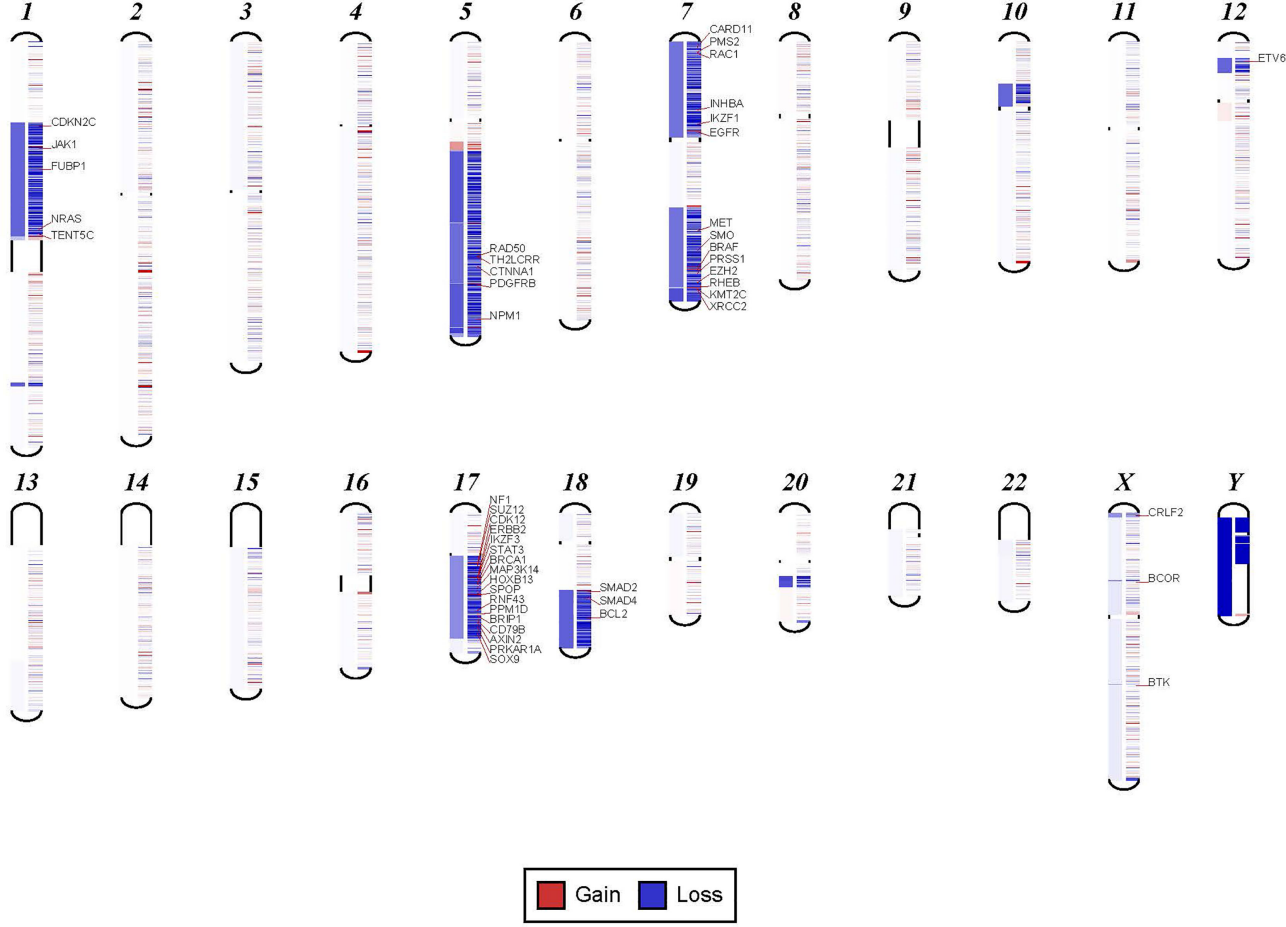
Examples in which cytogenetic complex findings can be significantly simplified by NGS analysis. Cytogenetic description for this sample states 43 to 45 chromosomes with the following abnormalities: “der(1)del(1)(p12p31)add(1)(q12),+3,add(3)(p11.2),add(3)(q11.2), del(5)(q13q34),add(6)(q21),-7,-10,del(10)(p13p15), der(11)t(11;17)(q23;q11.2),-12,-16,-17,add(17)(q11.2),add(18)(q21.1),add(18)(q21.3),-20,del(21)(q21q22),+3 4mar[cp13]/89 90,slx2[2]/46,XX[5]. NGS shows major abnormalities in 1p-, 5q-, -7, 10p-, 12p-, 17q-, 18q- and 20q-. However, in addition deletions of specific genes on chromosome X are also noted.

In order to confirm the clinical reliability of detected chromosomal structural abnormalities using liquid biopsy and a targeted NGS panel, we compared findings with conventional cytogenetics performed on BM samples. Eighty-nine liquid biopsy samples from AML or MDS patients had BM cytogenetic data obtained within two weeks from when the liquid biopsy sample was obtained. There were 33 samples with chromosomal and/or fusion abnormalities (37%), 3 samples (3%) with sole fusion abnormality, 8 samples (9%) with “no metaphases detected,” and 45 (51%) with normal karyotype (**Table 2**). Since the NGS panel is designed to detect only chromosomal gain or loss, the 4 cases with fusion genes were not detected by NGS (as expected). Three of the “no metaphases detected” cases had cytogenetic abnormalities detected by NGS and 5 showed no chromosomal gain or loss by NGS (**Table 2**). There was a difference in the description of the abnormalities as detected by cytogenetics when compared with those detected by NGS. **Table 2** illustrates examples of differences between findings reported by cytogenetics and findings by NGS testing. Simple abnormalities such as trisomies and monosomies were recognized in a similar fashion, but some complex findings such as isochromosomes, derivatives and dicentric chromosomes were described differently. One case showed tetraploid metaphases appeared as normal on NGS sequencing. NGS resolved the marker chromosomes that are described in the cytogenetic analysis. **Figure 2** shows an example of abnormalities described by cytogenetic analysis as “48,XY,del(5)(q13q33),+8,+mar[20]”. NGS showed the 5q deletion and 8q gain and gene amplification involving chromosome 11, particularly the KMT2A gene, which is most likely reported as a marker chromosome. In addition, trisomy 13 is noted by NGS that was not reported by cytogenetics. **Figure 3** shows an example in which cytogenetic complex findings can be significantly simplified by NGS analysis. The cytogenetic description states 43 to 45 chromosomes with the following abnormalities: “der(1)del(1)(p12p31)add(1)(q12),+3,add(3)(p11.2),add(3)(q11.2),del(5)(q13q34),add(6)(q21),-7,-10,del(10)(p13p15),der(11)t(11;17)(q23;q11.2),-12,-16,-17,add(17)(q11.2),add(18)(q21.1),add(18)(q21.3),-20,del(21)(q21q22),+3 4mar[cp13]/89 90,slx2[2]/46,XX[5]. NGS showed major abnormalities in 1p-, 5q-, -7, 10p-, 12p-, 17q-, 18q- and 20q-, as well as deletions of specific genes on chromosome X.

**Table 2:** Targeted NGS vs. Conventional karyotypic/cytogenetic testing in predicting myelodysplastic or acute myeloid leukemia risk stratification* in individual patient samples.

Sample #	CNV by Liquid Bx NGS	Cytogenetic report	Interpretation (agreement: Yes/No)
1	5q-, 7q-, 18p-, 19p+, 19q+, 21q+(amplification)	46 48,XX,-5,r(7),-18,add(19)(p13.1),der(21)t(5;21)(q13;p11.2),+ 2 5mar[cp16]/46,XX[4]	complex (Yes)
2	5q-, 7q-, -18 (bi-allelic 18p-), 19p+, 19q+, 21q+(amplification)	46 48,XX,-5,r(7),-18,add(19)(p13.1),der(21)t(5;21)(q13;p11.2),+ 2 5mar[cp16]/46,XX[4]	complex (Yes)
3	1q+, 7q-, 19q+, 21q+	46,XY,+1,der(1;7)(q10;p10)[2]/47,idem,+21[14]/48,idem,+8,+21[2] /47,idem,+8[1]/46,XY[1]	complex (Yes)
4	5q-, 8q+, and 17p-	46,XX,del(3)(q21q25),add(5)(p13),del(5)(q13q33),-17,der(17;21)(q10;q10),add(21)(p11.2),+mar[cp20]	poor (Yes)
5	5q-, 7q-, +8, 17p-, 17q1(proximal), 17q+(distal),-19, +21	46 47,XY,del(5)(q15q34),add(7)(q21),+8,del(11)(q22q23),-17,-19, -22,+2 3mar[15]/46,XY[5]	poor (Yes)
6	5q-; 7q-, 17p-, 18q-	45,XX,del(5)(q13q33),-7,der(17)add(17)(p11.1)add(17)(q23),del(1 8)(q21.3q23)[12]/45,XX,del(5)(q13q33),dic(7;17)(q11.2;p11.1),de l(18)(q21.3q23)[3]/46,XX[5]	complex (Yes)
7	2p-, 3p-, 5q-, 17p-, 17q-(partial), 19p+, 21q+	42 43,XY,add(3)(p13),-2,-3,-5,add(16)(q12.1),-17,add(19)(q13.1) ,-20,+2mar,inc[cp3] LIMITED STUDY	complex (Yes)
8	3p-, 5q-, 7q-, 12p- and +22.	45,X,-Y,add(1)(q21),del(3)(p13p25),dic(5;12)(q11.2;p11.2),add(7 )(q11.2),+mar[19]/46,XY[1]	complex (Yes)
9	monosomy 7	45, XX,-7[9]/46.xx[11]	poor (Yes)
10	5q-, +8, +11, +13, and 17q-	40 48,XY,+5,del(5)(q15q33),der(5;14)(p10;q10),der(5;17)(p10;q10 ),-7,+8,i(11)(q10),idic(13)(p11.2),add(14)(p11.2),add(16)(q24), i(17)(q10),-18,der(20)t(11;20)(q13;q13.3),-21,+22,+mar[cp17]/46 ,XY[3]	complex (Yes)
11	5q-, -7, 11q+, 12p-, 17p-, 18p-, 19q+	44,XY,-5,-7,inv(12)(p13q13),add(17)(p11.2),-18,der(19)ins(19);? (q13.1);?,-20,+2mar[8]/43,idem,-11,add(13)(q32)[5]/44,idem,r(11 )(p15q25)[2]/46,XY[7]	complex (Yes)
12	-5, 8p+, 9p-,11p-, 17p-, -18 and 20p-	45,XX,add(3)(q21),add(5) (q11.2)x2, der(6)t(6;17) (q27;q11.2),add(7) (q31),+8,der(13)t(5;13) (q15;q32),-17,1-18[10]/46,XX[4] INCOMPLETE STUDY	complex (Yes)
13	5q-, 8q+, 11p+(proximal amplification), 11q (KMT2A gene amplification), +13.	48,XY,del(5)(q13q33),+8,+mar[20]	poor (Yes)
14	3p-, 5q-, -7, and 12p-	43 44,XX,-3,dic(5;15)(q11.2;p11.2),-6,del(6)(p23p25),der(7;12)t (7;12)(p10;q10)ins(7);?(p11.2);?,+der()?t(?;3)(?;q12),+r[cp17]/ 46,XX[3]	complex (Yes)
15	4q-, 5q-, 7q-, +11, 12p-, 13p+, 13q+, -16, 17p-, -18, +21 and +Y.	44 50,XYY?c,-4,dic(5;17)(q13;p11.2),add(7)(q11.2),der(12)ins(12 );?(q13);?,-14,-21,-22,+2 8mar[cp14]/47 48,idem,+11,+13[cp3]/81 89,idemx2[2]/47,XYY?c[8]	complex (Yes)
16	7q-	46, XX, del(7)(q22q32)[19]/46,XX[1]	poor (Yes)
17	12p-	46,XY[20]	intermediate (Yes)
18	1q+, 2p+, 3q-, -4, -7, 9q+, 13q+, 17p-, 17q-, 20q+, 21q-	44 46,XY,add(2)(p11.2),-3,add(3)(q11.2),add(4)(q12),-5,der(5)t( 3;5)(p13;p13)ins(5);?(p13);?,del(6)(p23p25),-9,add(13)(q12),-17 ,-17,add(20)(q11.2),+3 5mar,inc[cp5] Limited Study	complex (Yes)
19	1q+ and trisomy 14.	46,XY,i(14)(q10)[20]	intermediate (Yes)
20	8p-, 9p- (PAX5, CD174, CDKN2A/B), 17p- and gain:1p+, 17p+.	45,XY,der(8)t(1;8)(q12;p21),inv(8)(p11.2q24.3),add(9)(p13),i(17 )(q10),-20[4]/46,XY[20]	poor (Yes)
21	1p-, 5q-, -7, 10p-, 12p-, 17q-, 18q- and 20q-	43 45,XX,der(1)del(1)(p12p31)add(1)(q12),+3,add(3)(p11.2),add(3)(q11.2), del(5)(q13q34),add(6)(q21),-7,-10,del(10)(p13p15), der( 11)t(11;17)(q23;q11.2),-12,-16,-17,add(17)(q11.2),add(18)(q21.1 ),add(18)(q21.3),-20,del(21)(q21q22),+3 4mar[cp13]/89 90,slx2[2 ]/46,XX[5]	complex (Yes)
22	3q-, 5q-, 7p-, 8q+, 12p-, 12q-, -16, 17p-, -18, -20	43,XY,del(5)(q13q33),del(7)(p13p22),add(9)(q13),der(12)add(12)(p11.2)del(12)(q14q21),-16,-18,-20[20]	complex (Yes)
23	+8 and 10q-	46,XY,der(4)t(4;8)(q33;q13),t(8;21)(q22;q22)[7]/47,XY,+8,t(8;21)(q22;q22)[5], LIMITED ANALYSIS	intermediate (Yes)
24	3p-, 5q-, -7, +8, 16q-, 17p-, 18q-, +21	44,XX,-3,del(5)(q15q34),r(7),+8,-16,add(17)(p11.2),-18,add(21)( q22)[20]	complex (Yes)
25	Trisomy 21	47,XX,+21[13]/46,XX[3]	intermediate (Yes)
26	8p+, 18p-	47,XY,+8[4]/46,XY[16]	intermediate (Yes)
27	1q+, 8q+	47,XY,dup(1)(q11q44),+8[17]/46,XY[3]	intermediate (Yes)
28	1p+, 5q-, +6, 7q-, -11, 17p- and others	44,XY,add(2)(p11.2),der(5)t(5;17)(q15;q21),add(6)(p21.3),del(7) (q22q36),-11,del(13)(q12q14),-14,der(16)t(14;16)(q24;q11.2),-17 ,add(21)(q22),+r[19]/46,XY[1]	complex (Yes)
29	monosomy 7 and 12p-	46,XY,r(7)[3]/46,XY[1], LIMITED ANALYSIS	poor (Yes)
30	Normal	92,XXXX,add(12)(q24.1)x2[20]	intermediate (Yes)
31	9p-	46,XY,del(9)(q13q34)[3]/46,XY[17]	intermediate (Yes)
32	2q+, 2q-(distal, IDH1 and ERBB4 deletion), 3p-, 5q-, 7q-, and 12p-.	39 45,XX,add(2)(q22),del(3)(p11),dic(5;7)(q11.2;q11.1),-12,-17, +mar[cp19]/46,XX[1]	complex (Yes)
33	5q+ (gain)	46,XX[20]	intermediate (Yes)
34	9p-(deletion of CDKN2A and CDKN2B)	NO METAPHASES DETECTED	poor (N/A)
35	7q- and 8q+	NO METAPHASES DETECTED	poor (N/A)
36	12p-	NO METAPHASES DETECTED	intermediate (N/A)
37	normal	No Metaphases Detected	intermediate (N/A)
38	Normal	NO METAPHASES DETECTED	intermediate (N/A)
39	Normal	NO METAPHASES DETECTED	intermediate (N/A)
40	Normal	No Metaphases Detected	intermediate (N/A)
41	Normal	NO METAPHASES DETECTED	intermediate (N/A)
42	Normal	46,XX,t(9;22)(q34;q11.2)[4]/46,XX[16]	no loss/gain (Yes)
43	Normal	46,XX,inv(16)(p13.1q22)[22]	no loss/gain (Yes)
44	Normal	46,XY,t(9;22)(q34;q11.2)[20]	no loss/gain (Yes)
	45 sample with no chromosomal abnormalities	45 samples with Normal Karyotype	45 sample (Yes)

*As per European Leukemia Network guidelines (13, 14).

CNV, copy number variations; Bx, biopsy.

To compare the two approaches, the cytogenetic and chromosomal findings of the 89 cases were grouped into three myeloid risk groups as per MDS and AML classification: poor, intermediate, and complex (5). This classification showed 100% concordance between the NGS chromosomal structural analysis and cytogenetic data.

## Discussion

We report here that targeted NGS testing of liquid biopsies can adequately detect mutations and chromosomal alterations in patients suspected of having myeloid and lymphoid neoplasms. Recently, complete genomic sequencing has been shown to be as accurate as conventional cytogenetic analysis, if not more sensitive (8).

In our study, using targeted NGS, we show that when neoplastic DNA is detected upon analyzing cfDNA from patients with myeloid and lymphoid neoplasms, chromosomal structural abnormalities can be demonstrated in 59% of patients with myeloid neoplasms and in 41% of patients with lymphoid neoplasms. We show that a neoplastic clone with VAF of 13% or more is adequate to define the sensitivity in detecting chromosomal structural abnormalities associated with AML and MDS. We found a 100% concordance rate between cytologic and cfDNA specimens in 89 AML or MDS patients when stratifying patients into intermediate, poor, or complex risk based on karyotypes. The data from this study supports the use of liquid biopsy and targeted NGS testing in detecting chromosomal structural abnormalities, specifically in myeloid neoplasms.

In addition to its sensitivity and high concordance with conventional BM cytogenetic testing, this technique has the added advantage of a short turnaround time of five to seven days, as compared to averages of seven to twenty-one days for conventional karyotypic analysis (6). The use of liquid biopsy may also allow for a less invasive approach to serial monitoring of patients undergoing treatment as it allows for evaluation without the need for repeat BM biopsies. Our technique also has the advantage of not relying on the presence of adequate metaphases in order for analysis to be done, with up to 19% of BM cytogenetic studies having inadequate number of metaphases or no growth (20). Furthermore, evaluation of chromosomal structural abnormalities by NGS can resolve some of the chromosomal structures detected by karyotyping such as marker chromosome and double minute chromosome. Obtaining adequate metaphases for karyotyping is also a significant problem, especially in patients with slow proliferating tumors such as CLL. NGS-based evaluation of chromosomal abnormalities can overcome this problem. The use of liquid biopsy is particularly useful in such cases when there are too few circulating tumor cells and a BM sample is not available.

Evaluating chromosomal gain or loss using targeted NGS was possible in this study due to the use of a relatively large number of genes (see **Table S1 in Supplement**) to cover important chromosomal regions that are usually involved in oncogenesis. These genes were selected because they are involved in oncogenesis rather than because they are relevant to covering chromosomal regions. Therefore, these genes provided an important mutation profile in addition to detecting chromosomal abnormalities. Smaller panels may not cover all chromosomal regions that are important in hematologic neoplasms. The ability of such a panel to detect both mutations and chromosomal abnormalities is a highly cost-effective and clinically useful approach in evaluating molecular abnormalities in hematologic neoplasms.

When compared to other new techniques for genetic analysis of patients with AML or MDS such as that of whole genome sequencing, our technique is as sensitive and reliable with a comparable turnaround time and the added benefit of not requiring a BM sample to obtain tumor genetic information (8). One of the advantages of conventional karyotyping is positive selection of the leukemic cells in the culturing process, which leads to enrichment of the leukemic cells. This enrichment is particularly relevant when the leukemic cells are limited in the analyzed sample; however, this has the potential to introduce bias by selecting subclones with more aggressive biology. This bias is eliminated when NGS is used for evaluating chromosomal structural abnormalities. Other methods for evaluating chromosomal structural abnormalities, such as optimal genome mapping, provide some advantages due to its objectivity, accuracy, and elimination of the need to culture samples. However, optimal genome mapping has limitations when DNA is degraded such as in cfDNA. More importantly, NGS analysis provides information at a higher resolution that can be at the gene level and even at the exon level in addition to providing information on mutations. NGS testing is needed for mutation analysis, and it is significantly more efficient and more cost effective to design the NGS assay to capture cytogenetic abnormalities as well.

Limitations of this study include a small sample of patients, single center design, and the inability to detect chromosomal translocations and fusion genes with the targeted NGS panel utilized. Furthermore, a recent study has shown that when mutation profiles from targeted NGS of cfDNA from peripheral blood were compared to those of BM, the concordance of these methods was high, but not identical (17). Our study did not evaluate this aspect and larger studies are needed to further corroborate our findings. The design of the targeted NGS panel can be expanded to cover chromosomal translocations. Additionally, RNA sequencing can be added for detecting fusion genes. The role of RNA sequencing in liquid biopsies is evolving and has the potential to provide information not only of detecting fusion abnormalities but also of expression profiles for various myeloid and lymphoid specific markers that can help in immunophenotyping.

## Conclusions

In conclusion, this data shows that liquid biopsy using targeted NGS is reliable in detecting chromosomal structural abnormalities in myeloid neoplasms. In specific circumstances, targeted NGS may be reliable and efficient to provide adequate information without the need for BM biopsy considering broad mutation profiling can be obtained through adequate sequencing within the same test. Overall, this study supports the use of liquid biopsy for early diagnosis and monitoring of patients with myeloid neoplasms.

## Data Availability Statement

The data presented in the study are deposited in the NCBI's Sequence Read Archive (SRA) repository, accession number PRJNA837911.

## Ethics Statement

The studies involving human participants were reviewed and approved by John Theurer Cancer Center and Hackensack Meridian Health. Written informed consent for participation was not required for this study in accordance with the national legislation and the institutional requirements.

## Author Contributions

MA, AI, AD, ID, JE, WM performed the research. MA, AI, JM, AG, AP, and JK designed the study. WM, MA, and AI analyzed the data. MA, AI, AD, and GK wrote the paper. MA and AI supervised the study and revised the paper. All authors read and revised the manuscript, and all authors have approved the submitted version.

## Conflict of Interest

Authors JE, ID, WM, and, MA are employed by Genomic Testing Cooperative. AG: Consultancy: COTA, KitePharma, Acerta, Xcenda, AstraZeneca; Research Funding: Bayer, CALGB, Constellation, Genentech/Roche, Infinity, Karyopharm, Morphosys, AbbVie, MD Anderson, Infinity Verastem, AstraZeneca, Celgene, Janssen, KitePharma, University of Nebraska. AI: Consultancy, Honoraria and/or advisory committee: TG Therapeutics, AstraZeneca. JM: Consultancy: AbbVie, CTI BioPharma, Jazz Pharmaceutical, Pfizer, Takeda and Novartis; Speaker’s bureau: Amgen, Bristol Myers Squibb, Incyte, Jazz Pharmaceuticals, Stemline, and Takeda; Equity ownership: COTA. JK: Speaker bureau: Bristol Myers Squibb. AP: Equity ownership: COTA, Genetic testing cooperative; Board of Directors or advisory committee membership: Genetic testing cooperative.

The remaining authors declare that the research was conducted in the absence of any commercial or financial relationships that could be construed as a potential conflict of interest.

## Publisher’s Note

All claims expressed in this article are solely those of the authors and do not necessarily represent those of their affiliated organizations, or those of the publisher, the editors and the reviewers. Any product that may be evaluated in this article, or claim that may be made by its manufacturer, is not guaranteed or endorsed by the publisher.
